# Circulating CCDC3 as an Indicator of Visceral Fat Accumulation in Patients with Type 2 Diabetes Mellitus

**DOI:** 10.3390/metabo16020111

**Published:** 2026-02-03

**Authors:** Lin Zhu, Xiaodie Fan, Jiangang Lu, Yutao He, Youyuan Gao, Sirong He, Longbin Lai, Ruobei Zhao, Rui Cheng, Xi Li, Fengning Chuan, Bin Wang

**Affiliations:** 1 Department of Medical Experimental Technology, College of Basic Medicine, Obesity and Metabolic Diseases Research Center, Chongqing Medical University, Chongqing 400016, China; 2 Department of Endocrinology, Chongqing University Fuling Hospital, Chongqing 408099, China; 3 Department of Nephrology, Chongqing University Fuling Hospital, Chongqing 408099, China; 4 Department of Immunology, College of Basic Medicine, Chongqing Medical University, Chongqing 400016, China

**Keywords:** CCDC3, visceral fat area, abdominal obesity, type 2 diabetes mellitus, circulating biomarker

## Abstract

**Background**: Visceral fat plays a central role in cardiometabolic risk among people with type 2 diabetes mellitus (T2DM), yet its assessment in routine clinical practice remains largely dependent on imaging techniques or indirect anthropometric measures. Identifying accessible blood-based markers that reflect visceral adiposity may facilitate improved phenotyping in this population. This study aimed to investigate whether circulating coiled-coil domain–containing protein 3 (CCDC3) reflects visceral fat accumulation in adults with T2DM. **Methods**: Public RNA-sequencing datasets and human adipose tissue samples were analyzed to identify CCDC3 as a visceral fat–enriched secretory gene. In this cross-sectional study of 160 adults with T2DM undergoing dual-energy X-ray absorptiometry, plasma CCDC3 was measured by ELISA. Associations between plasma CCDC3 and visceral fat area (VFA) were examined using multivariable regression. Logistic regression models for abdominal obesity (VFA ≥ 100 cm^2^), with and without CCDC3, were evaluated using receiver operating characteristic (ROC) analysis, calibration curves, decision curve analysis (DCA), and Shapley additive explanations (SHAP). **Results**: Circulating CCDC3 levels were positively associated with VFA (β = 3.11, *p* < 0.001), independent of demographic and metabolic factors. Incorporating CCDC3 into the baseline model significantly improved discrimination of abdominal obesity (AUC 0.820 vs. 0.663; *p* = 0.009). Calibration curves and DCA supported better model fit and higher net clinical benefit with CCDC3. SHAP analysis showed that CCDC3 contributed the greatest incremental importance beyond waist circumference, sex, and age. **Conclusions**: Circulating CCDC3 may serve as a blood-based biomarker reflecting visceral adiposity in adults with T2DM and provides complementary information beyond traditional anthropometric measures.

## 1. Introduction

Abdominal obesity (AO), characterized by the pathological expansion of visceral adipose tissue (VAT), is a critical driver of type 2 diabetes mellitus (T2DM) pathogenesis [1,2]. Excess VAT disrupts metabolic homeostasis via chronic inflammation, ectopic lipid deposition, and dysregulated adipokine secretion, collectively exacerbating insulin resistance and β-cell dysfunction [3,4,5]. Epidemiological studies consistently show that visceral adiposity, independently of body mass index (BMI), carries a 3- to 5-fold higher risk of T2DM than subcutaneous adipose tissue (SAT) [6,7], underscoring VAT’s unique pathogenic role and the clinical need for biomarkers that specifically reflect visceral fat burden and its metabolic consequences.

Current approaches to estimate visceral adiposity have notable limitations. Anthropometric measures such as waist circumference or waist-to-hip ratio lack specificity because they cannot distinguish VAT from SAT [8]. Visceral fat area (VFA) assessed by imaging is a superior surrogate: according to the 2024 Chinese Guidelines for the Clinical Management of Obesity, a VFA ≥ 100 cm^2^ defines AO in Chinese patients [9]. However, advanced modalities like dual-energy X-ray absorptiometry (DXA) and computed tomography (CT), although accurate, are costly, involve radiation exposure, and are impractical for routine use [10]. Moreover, these structural assessments do not capture VAT’s dynamic endocrine function, which actively secretes bioactive factors driving metabolic disease.

Adipose tissue functions as an endocrine organ by secreting a range of bioactive peptides and proteins, collectively referred to as adipokines [11,12]. Classical adipokines such as leptin and adiponectin reflect total adiposity or insulin sensitivity but lack VAT specificity [13]. Leptin is predominantly released from SAT, correlating with overall fat mass rather than visceral fat [14], while adiponectin, despite its inverse relationship with metabolic risk, shows no depot-specific expression [15]. Emerging proteomic studies reveal distinct secretory profiles between VAT and SAT, with VAT favoring pro-inflammatory cytokines and lipotoxic mediators [16]. Despite these advancements, most proposed biomarkers either lack reproducible clinical validation or show overlapping expression across fat depots, limiting their utility in distinguishing visceral from subcutaneous adiposity. The absence of a VAT-selective circulating biomarker hinders personalized risk stratification and targeted therapeutic interventions for AO-associated metabolic disorders.

Among candidate adipokines with depot-specific expression, CCDC3 (Favine) has recently emerged as a promising secretory protein enriched in visceral fat. Previous studies have linked CCDC3 to visceral adiposity, lipid metabolism regulation, and NF-κB–mediated inflammation inhibition, suggesting endocrine-like effects relevant to VAT biology [17,18,19]. These findings raise the possibility that circulating CCDC3 may serve as a biologically plausible blood-based signal of visceral fat burden in T2DM.

To test this hypothesis, we conducted a two-stage study integrating transcriptomic discovery and clinical validation. We first identified CCDC3 as a VAT-enriched secretory gene through bioinformatic screening of human adipose transcriptomes, and then examined its circulating levels and association with VFA measured by dual-energy X-ray absorptiometry in well-characterized adults with T2DM. We further evaluated whether CCDC3 improves the identification of abdominal obesity beyond conventional anthropometric indicators.

## 2. Materials and Methods

### 2.1. Microarray Data Collection and Bioinformatic Analysis

Microarray data from two datasets, GSE88837 and GSE58979, were downloaded from the National Center for Bioinformatics (NCBI) Gene Expression Omnibus (GEO) (http://www.ncbi.nlm.nih.gov/geo/, accessed on 26 January 2025) [20]. The GSE88837 dataset included visceral adipose tissue samples from 16 individuals with obesity (BMI ≥ 25 kg/m^2^, as defined by the WHO Western Pacific Region guideline) and 14 individuals without obesity (BMI < 25 kg/m^2^). The GSE58979 dataset comprised 45 samples from females with obesity, comprising 20 subcutaneous adipose tissue and 25 visceral adipose tissue samples. Detailed characteristics of the datasets are summarized in Table 1. Then, differential expression analysis was performed independently on GSE88837 and GSE58979 datasets using the “Limma” R package [21] in R 4.3.2 software. Genes with *p* values ≤ 0.05 and |log_2_ (fold change)| ≥ 0.585 were considered differentially expressed genes (DEGs). Subsequently, a list of significantly up-regulated genes was extracted separately. Finally, overlapping DEGs among GSE88837 and GSE58979 datasets, and secretory proteins were visualized using a Venn diagram created with the “venn” R package. Secretory proteins were obtained from the Human Protein Atlas database (https://www.proteinatlas.org/, accessed on 26 January 2025) [22]. A total of 3946 genes encoding secretory proteins were sourced from the protein class designated as “SPOCTOPUS predicted secreted proteins”.

### 2.2. Study Design and Participants

**Figure 1:**
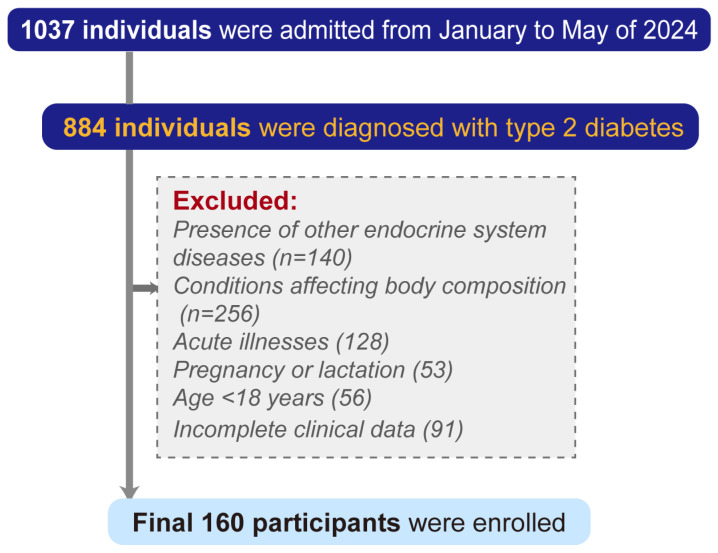
Flow diagram of participant selection. A total of 1037 individuals were admitted between January and May 2024, of whom 884 were diagnosed with T2DM. After excluding individuals with other endocrine diseases (n = 140), conditions affecting body composition (n = 256), acute illnesses (n = 128), pregnancy or lactation (n = 53), age less than 18 years (n = 56), or incomplete data (n = 91), 160 participants were included in the final analysis.

A cross-sectional study was conducted among patients with T2DM admitted to Chongqing University Fuling Hospital between January 2024 and May 2024. Inclusion criteria required patients to meet the 1999 World Health Organization (WHO) criteria for T2DM [23]. The exclusion criteria were as follows: (1) the presence of other endocrine disorders, including thyroid dysfunction, Cushing’s syndrome, hyperparathyroidism, and others; (2) conditions that may affect body composition, such as malignancies, immune system diseases, and diseases of the cardiovascular, cerebrovascular, liver, kidney, or pulmonary systems; (3) acute illnesses, including acute diabetic complications, infections, or trauma; (4) pregnancy or lactation; (5) individuals under the age of 18 years; and (6) incomplete clinical data. Ultimately, 160 participants were enrolled (Figure 1). The Ethics Committee of Chongqing University Fuling Hospital approved all the protocols described in this study. All the participants gave written informed consent.

### 2.3. Clinical Data Collection

Baseline demographic information, including age, sex, smoking and alcohol status, body mass index (BMI), and diabetes duration, was extracted from the electronic medical records. Additionally, values from a metabolic panel comprising fasting plasma glucose (FPG), glycated hemoglobin (HbA1c), lipid profile, serum uric acid (UA), estimated glomerular filtration rate (eGFR), urinary albumin-to-creatinine ratio (uACR), and indicators of nutritional status and systemic inflammation, such as hemoglobin, prealbumin, albumin, and high-sensitivity C-reactive protein (hsCRP), were obtained. Diabetes-related complications such as kidney disease, retinopathy, peripheral neuropathy, and carotid atherosclerosis, as well as other comorbidities including hypertension, coronary artery disease (CAD), and stroke, were documented. eGFR was calculated using the Chronic Kidney Disease Epidemiology Collaboration (CKD-EPI) formula. Hypertension was defined as systolic blood pressure ≥ 140 mmHg, diastolic blood pressure ≥ 90 mmHg, or current use of antihypertensive therapy. CAD included myocardial infarction, angina, or prior coronary revascularization. Stroke encompasses ischemic or hemorrhagic stroke.

Blood samples collected in EDTA vacutainers were centrifuged at 3000× *g* for 10 min to obtain plasma, which was subsequently stored at −80 °C until analysis. Plasma concentrations of Coiled-Coil Domain Containing 3 (CCDC3) were quantified using enzyme-linked immunosorbent assay (ELISA) kits (Ruixin Biological Technology Co., Ltd., Quanzhou, China) according to the manufacturer’s instructions.

### 2.4. VFA Measurement by DXA and Group

VFA was assessed using a whole-body DXA scanner (Discovery A, S/N 87352, Hologic, Bedford, MA, USA) equipped with Hologic APEX software (version 4.5.3) performed by a trained technician. The software automatically identifies the outer and inner margins of the abdominal wall on both sides of the DXA image based on fat and lean mass profiles within a 5 cm region across the abdomen, positioned 1 cm above the iliac crest. Total fat mass within this abdominal region, which includes both subcutaneous and visceral fat, is measured. Subsequently, the software quantifies subcutaneous fat between the skin line and outer abdominal wall on both sides and subtracts this estimate from total fat mass to calculate visceral fat area (VFA). These procedures are standardized and automated by Hologic [24].

Abdominal obesity (AO) was defined as VFA ≥ 100 cm^2^ according to guidelines from the Chinese Medical Association. Study participants were categorized into two groups based on VFA value: AO groups (VFA ≥ 100 cm^2^) and non-AO groups (VFA < 100 cm^2^).

### 2.5. Statistical Analysis and Interpretation of Models

Continuous variables are presented as mean ± SD or median (interquartile range) according to distribution and were compared between groups using Student’s *t* test or the Mann–Whitney *U* test as appropriate. Normality was assessed with the Shapiro–Wilk test and Q–Q plots. Categorical variables are summarized as counts (percentages) and compared using the χ^2^ or Fisher’s exact test.

Correlations between plasma CCDC3 and clinical parameters were examined using Pearson or Spearman coefficients. Multivariable linear regression was performed to assess the association between circulating CCDC3 concentrations and visceral fat area (VFA). Model 1 was unadjusted; Model 2 adjusted for age, sex, smoking, and alcohol use; and Model 3 further adjusted for diabetes-related factors, including diabetes duration, HbA1c, triglycerides (TG), low-density lipoprotein cholesterol (LDL-C), uric acid (UA), and high-sensitivity C-reactive protein (hsCRP).

Logistic regression was used to estimate odds ratios (ORs) for abdominal obesity (AO; per 1-SD increase in CCDC3). Discrimination was assessed using receiver operating characteristic (ROC) curves, and AUCs were compared by the DeLong test. Calibration was evaluated by the Hosmer–Lemeshow goodness-of-fit test and Brier score, with 1000 bootstrap resamples for internal validation. Decision curve analysis (DCA) quantified net clinical benefit across threshold probabilities.

To interpret the relative contribution of each variable to the predictive model, Shapley additive explanations (SHAP) analysis was performed using Python 3.11 (package shap v0.45). A linear SHAP explainer was applied under the assumption of feature independence. Global importance was ranked by the mean absolute SHAP value across all samples, reflecting each predictor’s average contribution to model output. Individual SHAP values were visualized to show the direction and magnitude of each variable’s effect on the predicted probability of abdominal obesity.

Effect modification by glycemic status was tested by adding a CCDC3×HbA1c interaction term (HbA1c as a continuous variable) to the fully adjusted model. HbA1c-stratified analyses (<6.5%, 6.5–<7.0%, 7.0–<8.0%, and ≥8.0%) were performed. Spearman’s correlation was used to assess the association between HbA1c and circulating CCDC3.

All analyses were conducted using GraphPad Prism 10.2 and R 4.3.2 (packages pROC, rms, and rmda). A *p* < 0.05 was considered statistically significant.

## 3. Results

### 3.1. Identification of CCDC3 as a VAT-Enriched Secretory Gene

**Figure 2:**
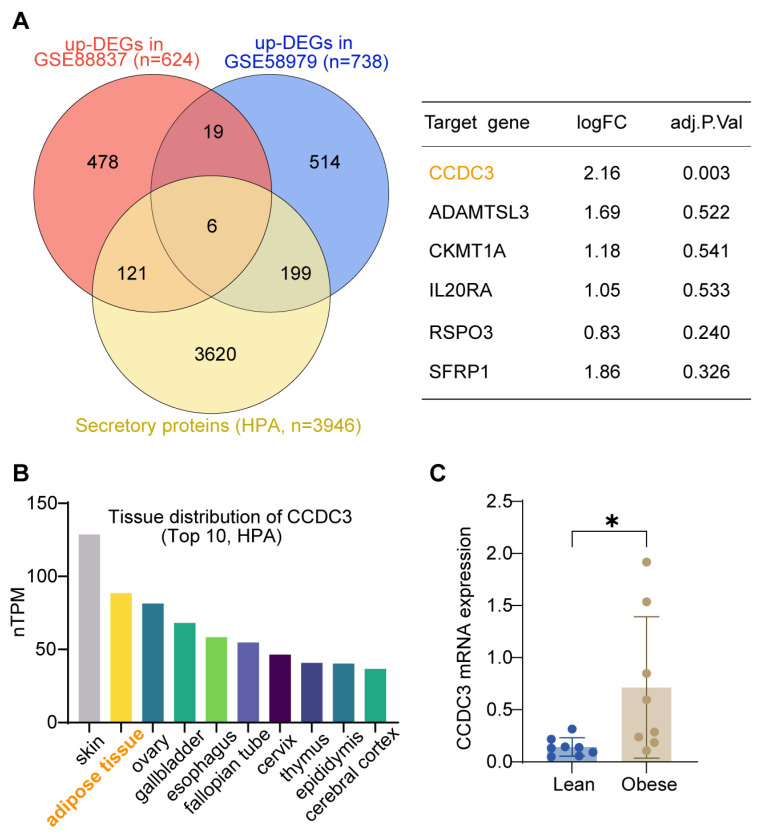
Identification and validation of CCDC3 as a VAT-enriched gene linked with obesity. (**A**) Venn diagram showing overlap among upregulated DEGs from both datasets and secretory proteins in the Human Protein Atlas (HPA), identifying six shared genes; CCDC3 exhibits the highest fold-change (log_2_FC = 2.16, adj. *p* = 0.003). (**B**) Tissue distribution of CCDC3 expression (top 10) based on nTPM from HPA, highlighting adipose tissue as a primary expression site. (**C**) qPCR validation of CCDC3 mRNA levels in human VAT from lean (n = 8) and obese (n = 8) subjects. Data are presented as mean ± SD; * *p* < 0.05 by Mann–Whitney U test.

Differential expression analysis identified 624 upregulated genes in GSE88837 and 738 in GSE58979 (|log_2_ fold change| ≥ 0.585 and *p* < 0.05). Among these, six genes overlapped between the two datasets and were also annotated as “secreted to blood” in the Human Protein Atlas (Figure 2A). CCDC3 showed the most tremendous fold change and was therefore selected for further investigation. Analysis of CCDC3 tissue distribution based on nTPM data from the HPA revealed adipose tissue as one of its top expression sites (Figure 2B). To confirm its relevance in obesity, CCDC3 mRNA levels were measured by qPCR in human visceral adipose tissue (VAT) from lean (n = 8) and obese (n = 8) subjects (Figure 2C).

### 3.2. Clinical Characteristics of Participants

Subsequently, 160 eligible participants (51.2% female) were enrolled, with a median age of 66 years, to examine the relationship between plasma CCDC3 levels and AO. The median duration of diabetes was 10.5 years, and the median HbA1c level was 9.2%. Participants were divided into two groups based on VFA value (AO groups: VFA ≥ 100 cm^2^, n = 100; non-AO groups: VFA < 100 cm^2^, n = 60). The main baseline characteristics of these groups are summarized in Table 1. Compared to the non-AO group, individuals with AO had higher BMI and waist circumference, elevated serum triglycerides (TG) and uric acid (UA), and lower eGFR and HDL-C levels. Albumin and hsCRP levels were also higher in the AO group, while other metabolic, nutritional, and clinical variables did not differ significantly.

### 3.3. Association Between Plasma CCDC3 Levels and VFA

**Figure 3:**
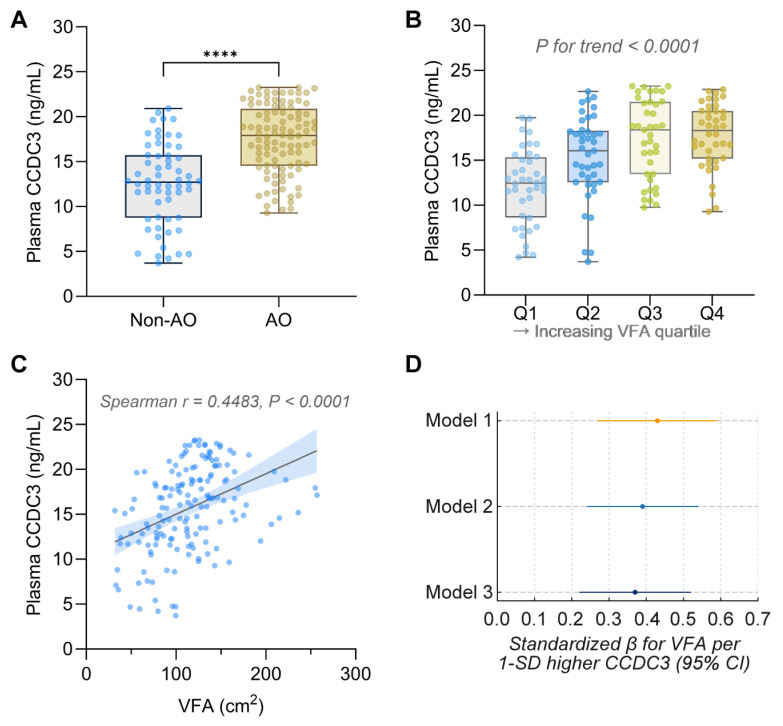
Association of Circulating CCDC3 with VAT in Patients with T2DM. (**A**) Plasma CCDC3 levels in participants with abdominal obesity (AO; defined as visceral fat area [VFA] ≥ 100 cm^2^) compared to those without (non-AO). Data are shown as box-and-whisker plots (displaying median, IQR, and range) with individual data points overlaid. **** *p* < 0.0001 by unpaired *t*-test with Welch’s correction. (**B**) Plasma CCDC3 levels across quartiles (Q1–Q4) of VFA, with group sizes varying due to tied values. One-way ANOVA indicated a significant difference among groups (F(3, 156) = 14.82, *p* < 0.0001). A significant positive linear trend was observed (β = 1.90 ng/mL per quartile increase, 95% CI 1.31–2.49; *p* < 0.0001). (**C**) Scatter plot showing the positive correlation between VFA and plasma CCDC3 levels. The solid line depicts the fitted linear trend, and the shaded area represents the 95% confidence interval. Spearman’s correlation coefficient r = 0.4483, *p* < 0.0001. (**D**) Multivariable linear regression analyses demonstrating the robust independent association between CCDC3 and VFA. Standardized β (per 1-SD increase in CCDC3) was 0.43 (95% CI 0.27–0.59) in Model 1 (unadjusted), 0.39 (0.24–0.54) after adjustment for age, sex, smoking, and alcohol, and 0.37 (0.22–0.52) after further adjustment for diabetes duration, HbA1c, triglycerides, LDL-C, uric acid, and hsCRP (*p* < 0.001 for all models).

Plasma CCDC3 levels were higher in patients with abdominal obesity compared to those without (Figure 3A). CCDC3 levels increased progressively across VFA quartiles (*p* for trend < 0.001; Figure 3B). CCDC3 showed a positive correlation with VFA (Spearman’s ρ = 0.448, *p* < 0.001; Figure 3C).

In multivariable linear regression (Figure 3D), higher CCDC3 remained independently linked to greater VFA. The standardized β was 0.43 (95% CI 0.27–0.59; *p* < 0.001) in the unadjusted model, 0.39 (0.24–0.54; *p* < 0.001) after adjusting for age, sex, smoking, and alcohol, and 0.37 (0.22–0.52; *p* < 0.001) after further adjustment for diabetes duration, HbA1c, triglycerides, LDL-C, uric acid, and hsCRP. Confidence intervals did not cross zero in any model, indicating a strong, independent positive association between circulating CCDC3 and visceral fat.

Effect modification by glycemic status was tested using a CCDC3×HbA1c interaction term in the fully adjusted model; the interaction was not significant (*p* for interaction = 0.710). HbA1c-stratified analyses showed broadly consistent effect directions across categories, with wider confidence intervals in the lower HbA1c groups due to smaller sample sizes (Supplementary Table S1). In addition, HbA1c was not significantly correlated with circulating CCDC3 (Spearman’s ρ = −0.120, *p* = 0.131; Supplementary Figure S1), indicating no strong association between glycemic control and circulating CCDC3 in this cohort.

### 3.4. Clinical Utility of Circulating CCDC3 in Abdominal Obesity

**Figure 4:**
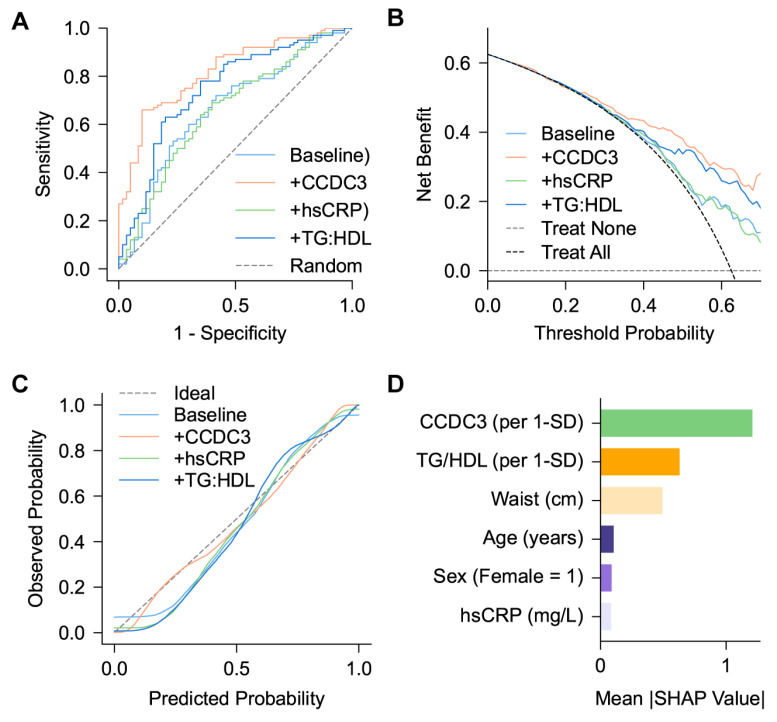
Discriminative and clinical utility of circulating CCDC3 for VAT in patients with T2DM. (**A**) Receiver operating characteristic (ROC) curves for the baseline model (age, sex, and waist circumference) and the baseline model plus circulating CCDC3. Adding CCDC3 enhanced discrimination for higher VAT (AUC 0.663 [95% CI 0.540–0.787] vs. 0.820 [95% CI 0.724–0.916]; ΔAUC = 0.157; *p* = 0.009, DeLong test). (**B**) Decision curve analysis (DCA) shows a greater net clinical benefit for the extended model (baseline + CCDC3) across different threshold probabilities. Gray dashed lines show the “treat all” and “treat none” strategies. (**C**) Calibration curves depicting agreement between predicted and observed probabilities of abdominal obesity. Dashed lines indicate the ideal reference; Hosmer–Lemeshow *p* values and Brier scores confirm good model fit after 1000 bootstrap resamples. (**D**) SHAP (SHapley Additive exPlanations) analysis illustrating each predictor’s relative contribution to the model output. Circulating CCDC3 and waist circumference were the most influential factors for higher visceral adiposity, followed by sex and age, highlighting the biological and statistical significance of CCDC3 as an independent indicator of VAT burden.

In logistic regression, each 1-SD increase in CCDC3 was linked to a higher likelihood of AO (adjusted OR 3.62, 95% CI 2.27–5.76; *p* < 0.001). Adding CCDC3 to the baseline model (age, sex, and waist circumference) significantly improved discrimination for AO (AUC 0.820 [95% CI 0.724–0.916] vs. 0.663 [0.540–0.787]; ΔAUC = 0.157, *p* = 0.009 by DeLong test) (Figure 4A).

Decision curve analysis (Figure 4B) showed a higher net clinical benefit for the extended model across a broad range of threshold probabilities, and calibration analysis indicated good agreement between predicted and observed probabilities (Hosmer–Lemeshow *p* = 0.74, lower Brier score; Figure 4C).

SHAP analysis further identified CCDC3 as the most influential factor in the model output, surpassing traditional anthropometric variables in predictive significance (Figure 3D).

## 4. Discussion

In this study, we identified circulating CCDC3 as a new biomarker indicating visceral fat in patients with T2DM. Transcriptomic analysis of public datasets showed that CCDC3 levels are significantly higher in visceral adipose tissue compared to subcutaneous fat [17]. In a well-characterized cross-sectional study population, plasma CCDC3 levels were markedly higher in individuals with abdominal obesity and increased progressively across quartiles of visceral fat area (VFA). The strong, independent association between circulating CCDC3 and VFA persisted even after adjusting for demographic and metabolic factors.

Adding CCDC3 to a basic model that includes age, sex, and waist circumference significantly improved the ability to detect abdominal obesity (AUC 0.820 vs. 0.663; ΔAUC = 0.157, *p* = 0.009). The expanded model also showed good calibration and provided a higher net clinical benefit according to decision curve analysis. SHAP analysis ranked CCDC3 as the most influential factor in the model, surpassing traditional anthropometric measures. Overall, these findings suggest that circulating CCDC3 offers additional value beyond standard metrics for identifying visceral fat accumulation in individuals with T2DM.

The tissue-specific expression pattern of CCDC3 supports these clinical observations. Ugi, S. et al. [17] found that CCDC3 mRNA is higher in omental fat than in subcutaneous fat and correlates with waist circumference in humans. Similar increases in epididymal white adipose tissue have been reported in obese and diabetic mice. Unlike classical adipokines such as leptin or adiponectin, which mainly reflect overall fat or systemic inflammation [25,26], CCDC3 shows depot-specific expression limited to visceral fat. This pattern supports its role as a visceral fat–derived factor that may link central adiposity to metabolic issues. Mechanistically, experimental data suggest that CCDC3 is involved in lipid metabolism and inflammation regulation through p63-related signaling and NF-κB inhibition [18,19,27], although further research is needed to clarify its exact functions in visceral fat changes.

From a clinical standpoint, measurement of plasma CCDC3 may provide a practical, non-radiologic, and cost-effective complement to imaging-based estimation of visceral fat. Waist circumference remains widely used but cannot distinguish visceral from subcutaneous fat [28], whereas CT and MRI—although accurate—are expensive and not feasible for routine screening [29]. DXA-derived VFA offers a validated surrogate that correlates strongly with MRI- and CT-based measurements [30,31]. Blood-based biomarkers such as CCDC3 could therefore facilitate population-level risk stratification and longitudinal monitoring in metabolic clinics. The integration of CCDC3 into clinical models may thus improve the identification of high-risk phenotypes among patients with T2DM.

There are several limitations. First, the cross-sectional design precludes causal inference between circulating CCDC3 and visceral fat accumulation. Second, although DXA provides validated estimates of VFA, it cannot fully distinguish visceral from subcutaneous compartments compared with CT or MRI. Third, our findings were derived from adults with T2DM and may not generalize to non-diabetic or multi-ethnic populations. While we found no evidence that HbA1c materially modified the CCDC3–VFA association, external validation in cohorts with a broader range of glycemic control is warranted. Future prospective studies with longitudinal follow-up, measures of insulin sensitivity, and cardiometabolic outcomes are needed to determine the prognostic significance of CCDC3.

## 5. Conclusions

The results of this study demonstrate that circulating CCDC3 is independently associated with visceral adiposity in patients with T2DM and enhances model performance for detecting abdominal obesity. These data highlight CCDC3 as a promising plasma biomarker linking visceral fat accumulation to metabolic risk and warrant further mechanistic and prospective validation.

## Figures and Tables

**Table 1:** Baseline clinical and metabolic features of patients with T2DM based on abdominal obesity (AO) status.

Parameters	Total (n = 160)	Non-AO (n = 60)	AO (n = 100)	*p* Value
Demographic characteristics				
Age (years)	66 (58–72)	64 (58–73)	67 (58–72)	0.61
Female n (%)	82 (51.2)	30 (50.0)	52 (52.0)	0.81
BMI (kg/m^2^)	23.4 (21.5–25.7)	21.2 (19.7–23.3)	24.7 (22.9–26.8)	<0.001
Waist circumference (cm)	89.6 (83.2–96.3)	81.3 (77.4–85.7)	94.2 (89.0–99.5)	<0.001
Metabolic profile				
HbA1c (%)	9.2 (7.0–10.8)	9.3 (6.8–10.9)	8.9 (7.0–10.8)	0.61
eGFR (mL/min/1.73 m^2^)	96.7 (73.1–121.8)	112.5 (81.9–123.9)	89.2 (66.0–114.9)	0.01
Uric acid (μmol/L)	297 (251–356)	281 (212–325)	305 (269–386)	0.002
Triglyceride (mmol/L)	1.26 (0.89–1.69)	0.98 (0.67–1.38)	1.34 (1.05–1.87)	<0.001
HDL-C (mmol/L)	1.14 (0.95–1.35)	1.25 (1.05–1.48)	1.05 (0.90–1.24)	<0.001
hsCRP (mg/L)	0.91 (0.45–2.29)	0.52 (0.34–1.76)	0.93 (0.56–2.90)	0.007
Diabetes-related characteristics				
Diabetes duration (years)	10.5 (6.0–17.0)	10.0 (6.0–16.8)	11.5 (4.3–17.0)	0.89
Diabetic retinopathy n (%)	49 (30.6)	20 (33.3)	29 (29.0)	0.56
Diabetic kidney disease n (%)	39 (24.4)	10 (16.7)	29 (29.0)	0.08
Hypertension n (%)	93 (58.1)	29 (48.3)	64 (64.0)	0.05

Note: Values are presented as median (interquartile range) or n (%). Differences between AO (abdominal obesity, visceral fat area ≥ 100 cm^2^) and non-AO groups were tested using the Mann–Whitney U test for continuous variables and the χ^2^ test for categorical variables. Abbreviations: BMI, body mass index; HbA1c, glycated hemoglobin; eGFR, estimated glomerular filtration rate; HDL-C, high-density lipoprotein cholesterol; hsCRP, high-sensitivity C-reactive protein.

## Data Availability

Dataset available on request from the authors.

## Supplementary Materials

The following supporting information can be downloaded at: https://www.mdpi.com/article/10.3390/metabo16020111/s1, Figure S1: HbA1c and circulating CCDC3; Table S1: HbA1c-stratified association between circulating CCDC3 and visceral fat area (VFA) in patients with T2DM.
