# Supplementary material for: Circulating CCDC3 as an Indicator of Visceral Fat Accumulation in Patients with Type 2 Diabetes Mellitus
Source: Metabolites. 2026 Feb 3;16(2):111. doi: 10.3390/metabo16020111 (PMC12942216; doi:10.3390/metabo16020111)
Supplement: Supplementary file 1 [file metabolites-16-00111-s001.zip › metabolites-4116558-supplementary.pdf]

Supplementary materials

Table S1. HbA1c-stratified association between circulating CCDC3 and visceral fat area (VFA) in patients with T2DM

HbA1c stratum	n	β per 1 ng/mL CCDC3 (95% CI)	Standardized β (95% CI)	P value
<6.5%	23	3.25 (-2.77, 9.28)	0.30 (-0.26, 0.86)	0.268
6.5–<7.0%	15	3.71 (-3.56, 10.98)	0.48 (-0.46, 1.41)	0.267
7.0–<8.0%	22	1.03 (-2.19, 4.25)	0.11 (-0.23, 0.45)	0.503
≥8.0%	100	4.28 (2.92, 5.65)	0.50 (0.34, 0.66)	<0.001

Notes: Models within each HbA1c category were adjusted for age, sex, diabetes duration, TG, LDL-C, and hsCRP. β indicates the adjusted change in VFA per 1 ng/mL increase in circulating CCDC3; standardized β was calculated within each category. P for interaction was 0.710 for CCDC3×HbA1c (continuous HbA1c) in the fully adjusted model and 0.635 for CCDC3×HbA1c_group (joint test).

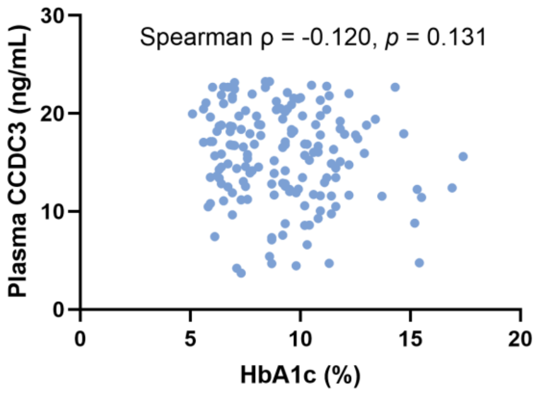

Figure S1. HbA1c and circulating CCDC3. Scatter plot of HbA1c (%) versus serum CCDC3 (ng/mL) in the study cohort (n = 160). Spearman's correlation: ϱ = −0.120, P = 0.131.
